# Serum Bilirubin Concentrations and the Prevalence of Gilbert Syndrome in Elite Athletes

**DOI:** 10.1186/s40798-022-00463-6

**Published:** 2022-06-27

**Authors:** Jana Woronyczová, Miroslava Nováková, Martin Leníček, Miloš Bátovský, Emil Bolek, Renata Cífková, Libor Vítek

**Affiliations:** 1 Institute of Medical Biochemistry and Laboratory Diagnostics, General University Hospital in Prague and 1st Faculty of Medicine, Charles University, Na Bojišti 3, Praha 2, 12000 Prague, Czech Republic; 2 Sports Research Institute of the Czech Armed Forces, Prague, Czech Republic; 3 Slovak Army Sport Centre Dukla Banská Bystrica, Banská Bystrica, Slovak Republic; 4 Center for Cardiovascular Prevention, 1st Faculty of Medicine, Charles University, and Thomayer University Hospital, Prague, Czech Republic; 5 4th Department of Internal Medicine, General University Hospital in Prague and 1st Faculty of Medicine,, Charles University, Prague, Czech Republic

**Keywords:** Bilirubin, Gene predisposition, Gilbert syndrome, Elite athletes, Sports performance, *UGT1A1* gene promoter

## Abstract

**Objectives:**

Bilirubin is a potent endogenous antioxidant and immunomodulating substance, which is also implicated in both cell signalling and various metabolic pathways. Mild elevation of systemic bilirubin concentrations provides substantial protection against many diseases of civilization. Rare published reports have suggested that serum bilirubin might also be relevant to sports performance. The purpose of the current study was to evaluate serum bilirubin concentrations and the prevalence of Gilbert syndrome (GS) in elite athletes.

**Methods:**

The study was carried out in 536 consecutive healthy elite athletes and in 2594 individuals of the Czech post-MONICA study representing the general Czech population. Serum bilirubin concentrations, the prevalence of benign hyperbilirubinemia > 17 µmol/L (1 mg/dL, a phenotypic sign of GS), and a variant of the *UGT1A1* gene promoter responsible for GS manifestation in Caucasians (rs81753472) were evaluated in study subjects.

**Results:**

Compared to the general Czech population, significantly higher serum bilirubin concentrations were found in elite athletes (9.6 vs. 11.6 µmol/L, *p* < 0.001), both in men (11.3 vs. 12.6 µmol/L, *p* < 0.001) and women (8.3 vs. 10.5 µmol/L, *p* < 0.001). Furthermore, the prevalence of GS was also significantly higher in elite athletes (9.6 vs. 22%, *p* < 0.001) together with the tendency to higher frequencies of the genotypes (TA)_7/7_ and (TA)_6/7_ *UGT1A1*.

**Conclusion:**

Elite athletes have significantly higher concentrations of serum bilirubin, the most potent endogenous antioxidant substance known. Simultaneously, the prevalence of GS syndrome is also much higher in elite athletes, suggesting that a mild elevation of serum bilirubin might predispose to better sports performance.

**Supplementary Information:**

The online version contains supplementary material available at 10.1186/s40798-022-00463-6.

## Key Points

- Elite athletes have significantly higher serum concentrations of serum bilirubin.
- The prevalence of GS syndrome is much higher in elite athletes.
- Serum bilirubin concentrations correlate with concentrations of uric acid and albumin, the other endogenous antioxidants.

## Introduction

The beneficial effects of regular physical exercise on overall health status are well known and appreciated. However, vigorous and exhaustive training sessions, particularly on a long-term basis, are accompanied by increased oxygen consumption, leading to an overproduction of reactive oxygen and nitrogen species (RONS). In a feedback mechanism, this process is counteracted by improving the antioxidant defence system of the human body to mitigate the potentially harmful effects of overproduced RONS [1–3]. Based on discoveries in the last few decades, bilirubin was recognized as one of the most potent endogenous antioxidants [4], although for a long time (and still today) it had been believed to be an ominous sign of underlying liver diseases or in sport misinterpreted as a marker of exercise-induced liver injury [5, 6]. Bilirubin is directly linked to the antioxidant capacity of the human body, which, due to the existing biliverdin-bilirubin redox cycle, largely overcomes molar expectations [7]. In fact, unconjugated bilirubin was demonstrated to be 20 times more effective than Trolox, a vitamin E analogue, in preventing LDL oxidation [8], and even more surprisingly concentrations of bilirubin as low as 10 nmol/L were found to be effective in protecting neuronal cultures from oxidative stress induced by 10,000 times higher concentrations of hydrogen peroxide [9]. In addition, bilirubin, as a potent immunosuppressive compound [10], plays a substantial role in the protection of tissues against inflammatory damage [11]. Even more interestingly, bilirubin has been shown to have important signalling functions (for review see [12]) acting as a real endocrine molecule [13].

In the general population, serum bilirubin concentrations between 5 and 17 µmol/L are generally considered the physiological range. Epidemiologically, they have a bimodal, non-normal distribution, which is due to the fact that from about 2 to 12% of healthy people have Gilbert syndrome (GS), also known as benign hyperbilirubinemia [14]. Intravascular bilirubin homeostasis is controlled by the *UGT1A1* gene encoding for bilirubin UDP-glucuronosyl transferase, responsible for its hepatic elimination out of the body [15, 16], and this gene is congenitally underexpressed in subjects with GS. The major genetic variant responsible for GS in Caucasians is a TA insertion in the TATA box of the *UGT1A1* gene promoter region. Homozygosity for this variant [(TA)_7_TAA, designated as the UGT1A1*28 allele], defines genotypic GS. However, due to its low penetrance, phenotypic manifestation occurs only in about 50% of carriers [16]. Other factors affecting blood bilirubin concentrations include: gender, ethnicity, age, adiposity, smoking, and dietary habits—to mention only the most important ones [17]. Furthermore, several studies have reported increased serum bilirubin concentrations in athletes, with exercise-induced hemolysis and rhabdomyolysis being attributed as the most likely causes [18–25] (for review, see ref [26]). However, these studies were underpowered, and no attempt to determine the molecular basis of GS was made in any of them.

Due to convincing experimental and clinical data that demonstrate the beneficial biological activities of bilirubin reported in the last few decades, and also due to lack of valid epidemiological data on markers of bilirubin metabolism, we hypothesized whether bilirubin might be a competitive advantage in elite sport. Therefore, the aim of our study was to determine serum bilirubin concentrations in elite athletes, to also determine the prevalence of GS, as well as the frequency of the UGT1A1*28 allele among them, compared to the general Czech population. Furthermore, our objective was also to determine the total antioxidant status in a subset of elite athletes and to correlate it with their serum bilirubin concentrations.

## Methods

### Study Populations and Subject Involvement

A total of 536 consecutive elite athletes (Table 1, 61% male, 14–45 years) were included in the study who had visited either the Sports Research Institute or the 4th Department of Internal Medicine of the General University Hospital in Prague and 1st Faculty of Medicine between 2019 and 2021. The subjects were representatives of many different disciplines of sport (Table 2), and all were of Caucasian origin.

**Table 1:** Basic characteristics of the studied cohorts

		Athletes (*n* = 536)	General population (*n* = 2594)
Age (years)	All	21.4 ± 5.4	48 ± 11
	Males	21.7 ± 5.5	48.3 ± 10.9
	Females	21 ± 5.1	47.7 ± 11
BMI (kg/m^2^)	Males	23.1 ± 1.9	29.2 ± 5.1
	Females	21.4 ± 1.5	27.3 ± 6
Smoking (%)	All	0	44

Data expressed as mean ± SD

**Table 2:** Athletes by sport discipline

Sports type	Sport	Number of subjects	Sex distribution (male/female)
Speed endurance	Athletics (intermediate runs) Cycling (track) Swimming	60	36/24
Strength endurance	Flat water canoeing Rowing	143	99/44
Endurance	Athletics (long runs; walking) Biathlon Cross-country skiing Cycling (road; mountain biking) Nordic combination	222	122/100
Speed strength	Alpine skiing Athletics (throws; jumps) Athletics (short runs) Bobsleigh Speed skating Sport gymnastics Weightlifting	65	34/31
Combat	Boxing Fencing Judo Wrestling	15	12/3
Games	Basketball Tennis Volleyball	28	20/8
Other	Shooting	3	2/1

A total of 2594 people from the general population of the Czech Republic were used to compare the bilirubin concentration data obtained from the elite athlete study population. This general population subset was derived from the cross-sectional Czech post-MONICA study, conducted between 2015 and 2018, which consisted of 2594 randomly selected from the Czech general population (48% men, aged 25–64 years, Table 1). [27]

The whole study was carried out in accordance with the Helsinki Declaration of 1975, as revised in 1983. The athlete study was approved by the CASRI Ethics Committee (No. 6/1-6/8 2019). The protocol of the Czech post-MONICA study was approved by the Ethics Committee of the Institute for Clinical and Experimental Medicine and Thomayer Hospital, Prague, Czech Republic (No. G 14-08-04). All participants provided their informed consent.

Before recruitment, all athletes received detailed information about the study objectives, which were also included in the informed consent form. Research questions were developed due to frequent requests by athletes and their supporting teams to look for new markers predisposing persons for high sports performance. The participation of individuals in the general population was governed by the detailed protocols of the Czech post-MONICA study. [28]

### Blood Collection and Laboratory Analysis

In elite athletes, a venous blood sample was collected in the morning, while the athletes were fasting and analysed in the laboratory within 1 h of collection for standard biochemical (lipids, glucose, liver enzymes, urea, creatinine, iron metabolism) and hematological (complete blood count) parameters. An aliquot of serum was immediately stored at − 80 °C for later antioxidant analyses (TAS and GLUT RED, see below).

The determinations of serum biochemical and hematological parameters were performed on automatic analysers (UniCel DxC 800 Synchron Clinical Systems, Beckman Coulter, UK; Chemistry Analyser BS-240; and Haematology Analyser BC-3600, Mindray Bio-Medical Electronics, China, respectively).

For the determination of the prevalence of phenotypic GS, based on the upper limits of normal in our laboratories, the following values of liver function enzymes were considered abnormal: alanine aminotransferase (ALT) > 0.78 μkat/L and *γ*-glutamyl transferase (GGT) > 0.84 μkat/L.

### Determination of Total Antioxidant Status and Glutathione Reductase

Total antioxidant status (TAS) and glutathione reductase activity (GR) were determined in a subset of the athlete group (*n* = 183) using TAS and GLUT RED spectrophotometric kits, respectively, both according to the manufacturer’s instructions (Randox Laboratories Ltd., UK). These subjects were those consecutively examined at the Sports Research Institute of the Czech Armed Forces between 2019 and 2020.

### DNA Analysis

DNA analysis of (TA)_n_ *UGT1A1* gene promoter variants was performed in a subset of the elite athlete group, which consisted of 136 subjects (90 men and 46 females). Again, these subjects were those consecutively examined at the Sports Research Institute of the Czech Armed Forces between 2019 and 2020. Similarly, a subgroup randomly selected from the general population (*n* = 605, 279 males and 326 females) was used for comparison [27]. DNA was isolated from the whole EDTA K_3_ blood samples using a slightly modified method by Miller et al. [29]

The *UGT1A1* gene promoter variants were analysed by multicoloured capillary electrophoresis as previously described, with some slight modification (for primer sequences, see Additional file 1: Table S1) [30]. The number of (TA) repetitions in the *UGT1A1* gene promoter (dbSNP rs81753472) was determined by fragment analysis performed by SEQme (Dobris, Czech Republic).

### Statistical Analyses

Data are expressed as the mean ± SD, or as the median and IQ range when the data were non-normally distributed. The *T* test or the Mann–Whitney rank sum test was used to compare laboratory parameters. The frequency of the alleles was evaluated using the Chi-square test. ANOVA on ranks with Dunn's post hoc analysis was used for all pairwise comparisons in groups of different genotypes of the *UGT1A1* gene promoter. Linear regression analyses were used to compare the possible relationship between serum bilirubin concentrations and age, while logistic regression analyses were used to assess the predictive role of serum bilirubin concentrations in elite athletes. All analyses were performed with the alpha set to 0.05. Statistics were calculated using SigmaPlot v. 14.5 (Systat Software, Inc. CA, USA).

## Results

### Serum Bilirubin Concentrations and Prevalence of GS in Elite Athletes

Compared to the general Czech population [27], significantly higher serum bilirubin concentrations were found in elite athletes (9.6 vs. 11.6 µmol/L, *p* < 0.001), with this difference observed in both men (11.3 vs. 12.6 µmol/L, *p* < 0.001) and women (8.3 vs. 10.5 µmol/L, *p* < 0.001) (Table 3). Additionally, the prevalence of a phenotypic GS was also significantly higher in elite athletes (9.6 vs. 22%, < 0.001), and this striking difference was observed for both male (12.6 vs. 27.7%, < 0.001*)* and female athletes (6.7 vs. 13.3%, < 0.001) (Table 3)*.*

**Table 3:** Serum bilirubin concentration and prevalence of GS in elite athletes

		Athletes (*n* = 536)	General population (*n* = 2594)	*P*-value
Bilirubin (µmol/L)	All	11.6 (8.5–16)	9.6 (6.9–13.2)	< 0.001
	Males	12.6 (9.2–17.8)	11.3 (8.4–15.2)	< 0.001
	Females	10.5 (7.5–13.3)	8.3 (6.1–11.1)	< 0.001
Prevalence of GS phenotype (%)	All	22	9.6	< 0.001
	Males	27.7	12.6	< 0.001
	Females	13.3	6.7	< 0.001

For bilirubin concentrations, data expressed as median and IQ ranges

*GS* Gilbert syndrome

In logistic regression analysis, each micromolar increase in serum bilirubin concentration was associated with a 4.6% increase in the probability of being grouped with elite athletes, thus demonstrating in a different way the fact that bilirubin is significantly higher in athletes.

The higher average age of the control population as a possible confounding factor was not confirmed in linear regression analyses performed for entire control population (containing 2594 subjects with median age of 49 years, IQ range 39–58 years, and min–max age 25–65 years), as well as for men and women, in whom serum bilirubin concentrations were not at all affected by age.

No differences in serum bilirubin concentrations were found in athletes belonging to sports disciplines involving endurance/strength endurance or strength/speed endurance (data not shown).

### ***Association Between Serum Bilirubin Concentration and (TA)***_***n***_*** UGT1A1 Gene Promoter Variants in Elite Athletes Compared to the General Population***

The frequencies of the genotypes (TA)_7/7_ and (TA)_6/7_ in both male and female athletes did not differ from the general population (Table 4). Serum bilirubin concentrations, when evaluated for individual (TA)n *UGT1A1* promoter genotypes, were substantially higher in athletes compared to the general population (Table 4). As expected, serum bilirubin concentrations depended on the presence of the (TA)_7_ allele, both in athletes as well as in the general population (Table 4).

**Table 4:** Association between serum bilirubin concentration and (TA)_n_ *UGT1A1* gene promoter variants

*UGT1A1* genotype	Elite athletes (*n* = 136)				General population (*n* = 605)			
	Males (*n* = 90)		Females (*n* = 46)		Males (*n* = 279)		Females (*n* = 326)	
	*n*	Bilirubin	*n*	Bilirubin	*n*	Bilirubin	*n*	Bilirubin
(TA)_6/6_	^+^35 (38.9%)	9.8 (8.1–13)	21 (45,7%)	8.3^*^ (6.3–9.9)	121 (43.4%)	9.2 (6.7–11.5)	^++^145 (44.5%)	6.5 (4.7–9.1)
(TA)_6/7_	41 (45.6%)	14.0^*^ (10.5–17)	17 (37%)	10.4^*^ (8.5–13)	143 (51.3%)	11.3 (8.0–15.2)	151 (46.3%)	8.2 (6.2–10.4)
(TA)_7/7_	14 (15.6%)	28.6 (18.5–33.5)	8 (17.4%)	22.2^*^ (15.8–25.5)	38 (13.6%)	21.6 (14.6–28.4)	30 (9.2%)	14.6 (10.0–19.8)
*P*-value^**^		< 0.05		< 0.05				

Data expressed in µmol/L as median and IQ range. ^*^P < 0.05 for comparisons with the general population of the appropriate gender. ^**^ANOVA on ranks with Dunn's post hoc analysis was used for all pairwise comparisons

^+^Includes 2 individuals with genotype (TA)_5/6_. ^++^Includes 1 individual with genotype (TA)_5/6_

### Relationship Between Serum Bilirubin Concentrations with Other Laboratory Markers in the Athlete Group

**Fig. 1:**
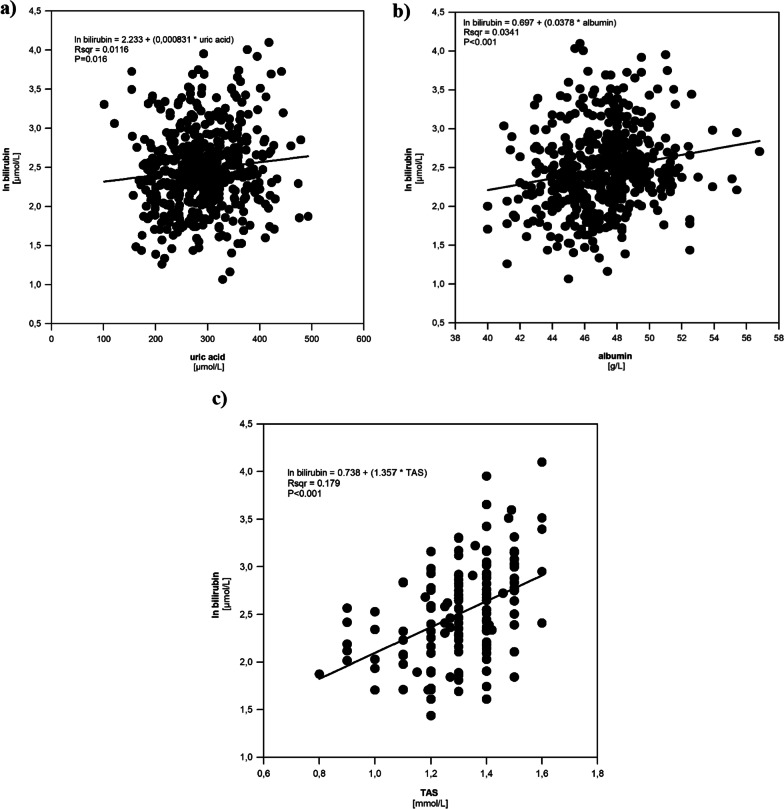
The association between serum bilirubin concentrations and **a** uric acid, **b** albumin concentrations, and **c** TAS in the athlete group. TAS, total antioxidant status

The possible relationship between serum bilirubin concentrations and standard metabolic laboratory markers was evaluated in the elite athlete group using linear regression analyses. While there was no association between serum bilirubin and glucose, urea, or cholesterol concentrations, a borderline negative association was observed for serum triacylglycerols (*p* = 0.058). A strong positive association of serum bilirubin concentrations was observed for uric acid and albumin concentrations (Fig. 1a, b), which themselves are potent and biologically relevant antioxidant substances. [31–33]

### Relationship Between Serum Bilirubin Concentrations and TAS and GR Activity in the Athlete Group

Serum TAS and GR activity were analysed in a subset of the athlete group (*n* = 183). The median serum TAS was 1.33 ± 0.15 mmol/L, while the median serum GR activity was 60 ± 7.7 U/L. A significant positive correlation was observed between serum bilirubin and serum TAS (*R*^2^ = 0.179, *p* < 0.001) (Fig. 1). GR activity was only slightly correlated with TAS (*R*^2^ = 0.035, *p* < 0.05); however, the correlation between serum bilirubin and GR activity was not present.

## Discussion

In our study, elite athletes were found to have significantly higher serum concentrations of serum bilirubin, the most potent endogenous antioxidant substance known [4]. Furthermore, the prevalence of GS was much higher in these elite athletes.

### Factors that Possibly Affect Serum Bilirubin Concentrations in Elite Athletes

#### Regular Training

It seems that regular physical exercise is associated with an increase in serum bilirubin concentrations, most likely as a feedback mechanism to regulate the increased oxidative stress that accompanies training processes. Our data are consistent with most published studies. In fact, serum bilirubin concentrations also increased significantly in response to regular soccer training [18], and similar effects have also been reported in other studies [19]. Some authors have suspected exercise-induced hemolysis and rhabdomyolysis, which is present primarily immediately after intense physical exercise [21–24], as demonstrated in studies on marathon and ultramarathon runners [21, 34]. However, the long-term bilirubin-elevating training effect is associated with an increase in other body antioxidant reserves, as well [19], indicating a rather positive protection feedback mechanism. Nevertheless, some contribution of increased bilirubin turnover due to exercise-induced hemolysis cannot be excluded [35]. This assumption is also corroborated by the positive correlation of serum bilirubin concentrations and TAS observed in our study. Interestingly, serum bilirubin concentrations are likely to be affected by the training/competition phase of the year, as shown in German Bundesliga soccer players [36], although this seasonal aspect was not investigated in our study.

On the other hand, not all published reports agree with any positive association of serum bilirubin and training. For example, no such effect was detected in a small study on soccer players and sedentary controls. [37]

#### Low Adiposity

Another factor that possibly contributes to higher systemic bilirubin concentrations in elite athletes is their much lower adiposity due to regular training, as also evidenced by much lower BMI values in athletes studied (Table 1). In fact, serum bilirubin concentrations in the general population are negatively correlated with body mass index [38, 39]. In obese subjects, bilirubin is most likely overconsumed due to obesity-induced oxidative stress [40]. In this context, notable data from a recent animal study revealed an exercise-induced increase in plasma bilirubin concentration, which was accompanied by substantial improvements in glucose and lipid metabolism [41]. These findings coincide with observations by Swift et al*. *[42], who documented significant increases in serum bilirubin concentration in previously sedentary postmenopausal women after being placed on an exercise regimen.

#### Smoking

Smoking is an important factor that decreases serum bilirubin concentrations due to increased oxidative stress [27]. As many as 44% of our subjects in the general population cohort were smokers [27], while virtually no smoker was present in our elite sport group (Table 1), suggesting that this might be a factor responsible for the dramatic difference observed in serum bilirubin concentrations between elite athletes and the general population. However, smoking in our cohort of the general population was responsible for only a 7% decrease in serum bilirubin concentrations [27], and if only non-smokers were included in the comparison, the difference in serum bilirubin concentrations would still have remained stunning (serum bilirubin in non-smokers in the general population was 9.9 µmol/L, IQ range 7.3–13.4 µmol/L). [27]

#### Age

Another important factor that could affect serum bilirubin concentrations is age, which differed significantly between athletes and the general population (Table 1). In fact, increasing age has been demonstrated to play a role in decreasing serum bilirubin concentrations in men (by 0.029 µmol/L with each year), but not in women in a large NHANES study [43]. However, this trend was not observed in our control population. Therefore, neither smoking status nor age substantially affected the large difference in serum bilirubin concentrations between athletes and the general population, as observed in our study.

### Prevalence of Phenotypic GS

In correspondence with significantly elevated serum bilirubin concentrations, the prevalence of phenotypic GS among our elite athletes reached 22%, which is even more than has been described in the few reports published to date. In fact, a prevalence of GS of 9% was observed in a recent Australian study [44], while a prevalence rate of almost 19% was reported in a Polish study on elite athletes, curiously equal in men and women [45]. Reports from Russia suggest a prevalence of GS among elite athletes in the range of 2–18.7% [46, 47], but these studies were not sufficiently described, and appear to suffer from substantial methodological issues, and thus cannot be considered reliable. What is more significant is that no attempts have been made in any of these studies to determine the molecular basis of GS.

### ***Association Between Serum Bilirubin Concentration and (TA)***_***n***_*** UGT1A1 Gene Promoter Variants***

For the first time, our study on elite athletes demonstrates the frequencies of (TA)_n_ *UGT1A1* gene promoter variants, responsible for the manifestation of GS in the Caucasian population. The frequencies of the (TA)_7/7_ and (TA)_6/7_ alleles did not differ between athletes and the general population (Table 4). However, serum bilirubin concentrations, when evaluated for individual (TA)_n_ *UGT1A1* promoter genotypes, were substantially higher in athletes compared to the general population (Table 4). There are two main factors that contribute to these observations. It seems that the (TA)_7/7_ genotype, to some extent, may predispose subjects to achieve superior performance in sport. However, the ability to increase serum bilirubin concentrations by regular training (with or without a (TA)_n_ *UGT1A1* predisposition) seems to be even more important.

### Oxidative Stress Defence in Elite Athletes

It is well known that strenuous exercise dramatically increases oxygen consumption in working muscles, leading to a marked increase in RONS formation [1]. Intense exercise induces an inflammatory response, and this is associated with increased oxidative stress and antioxidant activity [48]. Furthermore, increased antioxidant potential after exercise training has also been reported in other studies [19, 49–51]. According to the theory of hormesis, the generation of RONS in response to exercise preconditions skeletal muscle and other tissues by adapting their redox status to these multiple oxidative challenges, chronically elevating their antioxidant defence mechanisms [3]. Even more importantly, it seems that changes in redox status during strenuous exercise are more complex, representing virtually all essential mechanisms for muscle physiological and metabolic adaptation. [52]

Significantly higher serum bilirubin concentrations in elite athletes are directly related to serum TAS. Furthermore, positive correlations of serum bilirubin concentrations with serum concentrations of uric acid and albumin, two other potent antioxidants, were also found. These data suggest that upregulation of the antioxidant defence system in elite athletes is complex, and that bilirubin, indeed, may play both protective and beneficiary roles during and after exercise.

### Other Possible Implications of Mildly Elevated Serum Bilirubin Concentrations in Elite Athletes

It is important to emphasize that each micromolar change in serum bilirubin concentration is associated with a marked beneficiary modification of metabolic risk factors [53] as well as the risk of oxidative stress-related diseases of civilization [14, 54]. With the advent of new discoveries that bilirubin functions as a signalling and real endocrine molecule [12, 13], it is tempting to speculate that the higher observed serum bilirubin concentrations plus the prevalence of phenotypic GS in elite athletes, could provide substantial metabolic advantages (associated with exercise) to those with lower bilirubin levels.

## Limitations of the Study

Our study has several limitations. First, our control population differed substantially in many variables, including: age; anthropometric parameters; presence of concomitant diseases, either latent or apparent, which must have been present in our control subjects and which certainly may affect serum bilirubin concentrations; different lifestyle (more sedentary and other unhealthy lifestyle habits such as smoking, alcohol consumption, unhealthy food intake). Nevertheless, as discussed above, we have tried to eliminate possible confounding effects of these variables by additional sub-analyses. Other limitations include the fact that some analyses (genotyping, determinations of oxidative stress markers) could be performed only in a subset of the elite athlete group. Furthermore, it would be very interesting to correlate the parameters of bilirubin metabolism with the success rate of individual athletes, which, however, was beyond the scope of the current study.

## Conclusions

Our point in view on mild increases of serum bilirubin concentrations has changed dramatically over the last few decades. Although in the past and sometimes even to this day, individuals with GS were considered predisposed to chronic fatigue and various gastrointestinal problems. They were also advised against physical exercise or any appropriate improvements involving a training routine [6]; however, it is now clear that GS as well as any increase in serum bilirubin concentration (when not due to underlying liver disease or hemolysis), is likely to represent a selective advantage, due to the potent beneficial biological effects of bilirubin that probably result in even better sports performance.

## Supplementary Information

**Additional file1:** **Table S1. **Primers used in genotyping analyses.

## Data Availability

Original data (i.e. anonymised participant data) are available from the corresponding author upon a reasonable request.
