# Supplementary material for: Serum Bilirubin Concentrations and the Prevalence of Gilbert Syndrome in Elite Athletes
Source: Sports Med Open. 2022 Jun 27;8:84. doi: 10.1186/s40798-022-00463-6 (PMC9237193; doi:10.1186/s40798-022-00463-6)
Supplement: Supplementary file 1 — Additional file1: Table S1. Primers used in genotyping analyses. [file 40798_2022_463_MOESM1_ESM.docx]

**Additional file 1. Table S1. Primers used in genotyping analyses.**

| Forward primer | 5´ ‑ TGG TGT ATC GAT TGG TTT TTG C ‑ 3´ |
| --- | --- |
| First reverse primer | 5´ ‑ CAT CCA CTG GGA TCA ACA GTA TC ‑ 3´ |
| Second reverse primer | 5´ ‑ ACT ATT TCA TGT CCC CTC TGC TG ‑ 3´ |
| Third reverse primer | 5´ ‑ CGT CTT CAA GGT GTA AAA TGC TC ‑ 3´ |
| Fourth reverse primer | 5´ ‑ TCA AAA ACA TTA TGC CCG AGA C ‑ 3´ |

The forward primer was labelled at the 5′ end with either 6‑FAM, NED, or VIC fluorescent dyes (Thermo Fisher Scientific, Czech Republic).
